# Successful recovery of COVID-19 pneumonia in a patient from Colombia after receiving chloroquine and clarithromycin

**DOI:** 10.1186/s12941-020-00358-y

**Published:** 2020-04-24

**Authors:** José Millán-Oñate, William Millan, Luis Alfonso Mendoza, Carlos Guillermo Sánchez, Hugo Fernandez-Suarez, D. Katterine Bonilla-Aldana, Alfonso J. Rodríguez-Morales

**Affiliations:** 1 Infectious Diseases Division, Centro Médico Imbanaco, Cali, Valle del Cauca Colombia; 2 Asociación Colombiana de Infectología, Bogotá DC, Colombia; 3 Fundación Hospital San Jose de Buga, Buga, Valle del Cauca Colombia; 4 Radiology Division, Centro Médico Imbanaco, Cali, Valle del Cauca Colombia; 5 Incubator in Zoonosis (SIZOO), Biodiversity and Ecosystem Conservation Research Group (BIOECOS), Fundación Universitaria Autónoma de las Américas, Sede Pereira, Pereira, Risaralda Colombia; 6 Public Health and Infection Research Group, Faculty of Health Sciences, Universidad Tecnológica de Pereira, Pereira, Risaralda Colombia; 7 Grupo de Investigación Biomedicina, Faculty of Medicine, Fundación Universitaria Autónoma de las Américas, Pereira, Risaralda Colombia

**Keywords:** Coronavirus Disease 2019 (COVID-19), Severe Acute Respiratory Syndrome Coronavirus 2 (SARS-CoV-2), Chloroquine, Colombia, Latin America

## Abstract

**Background:**

COVID-19 pandemics is a challenge for public health and infectious diseases clinicians, especially for the therapeutical approach that is not yet adequately defined. Amid this situation, investigational agents are being used, including chloroquine. We report here the clinical features and therapeutic course of the first reported patient with confirmed COVID-19 pneumonia that recovered in Colombia, after the use of chloroquine and clarithromycin.

**Case presentation:**

A 34-year-old male, returning from Spain, presented with complaints of fever, and cough, and class-II obesity, being hospitalized. The respiratory viruses and bacteria tested by FilmArray^®^ PCR were negative. Two days later, clarithromycin was started because the patient was suspected as community-acquired pneumonia. At the third day, the rRT-PCR confirmed the SARS-CoV-2 infection. A day later, chloroquine was started because of that. His chest computed tomography was performed and showed bilateral multifocal ground-glass opacities with consolidation, which suggested viral pneumonia as a differential diagnosis. Progressively his clinical condition improved and at day 9, patient rRT-PCR for SARS-CoV-2 became negative. The patient was discharged and isolated at home per 14 days.

**Conclusions:**

Our patient improved significantly. This and other COVID-19 cases are urgently demanding results from clinical trials that support evidence-based therapeutical approaches to this pandemic and the clinical management of patients, especially those at critical care.

## Background

In late February 2020, the pandemic of the Coronavirus Disease 2019 (COVID-19), caused by the Severe Acute Respiratory Syndrome Coronavirus 2 (SARS-CoV-2), originated 2 months earlier in China, arrived in Latin America [1–3]. In this region, all the countries in South America, have been so far affected with imported cases (since March 2020), mainly from Italy and Spain [2]. After the identification of cases in Brazil and Mexico, other countries in the region begun to report confirmed COVID-19 cases, including Colombia [4, 5].

The first case in Colombia was confirmed, by real-time reverse-transcription–polymerase-chain-reaction (rRT-PCR), on March 6, 2020, in the capital Bogota [5]. After that case imported from Italy, in just 16 days, a total 231 cases have been confirmed in 38 municipalities in 19 departments across the country (March 22, 2020), including Valle del Cauca department, the third most populated of Colombia (4852,896 inhabitants, 2020; Colombia 49,834,727). On March 9, 2020, 3 days after the first case of Colombia, the second confirmed case was diagnosed at Buga municipality, Valle del Cauca, proceeding from Spain.

Right now, there many clinical and therapeutical concerns regarding COVID-19, in addition to the epidemiological and public health issues that represent the ongoing pandemic. Many investigational agents are being explored for antiviral treatment of COVID-19, and enrollment in clinical trials should be discussed with patients or their proxies [6–9].

Specific investigational agents have been described in some limited observational series or are being used anecdotally based on in vitro or extrapolated evidence, even including in some interim guidelines [9–12]. It is essential to acknowledge that no good controlled data are supporting the use of any of these agents, except for a recent randomized, controlled, open-label trial involving hospitalized adult patients with confirmed SARS-CoV-2 infection, that showed no benefit with lopinavir-ritonavir (LPV/RTV) treatment beyond standard care [13]. Nevertheless, in the modified intention-to-treat analysis, the between-group difference in the median time to clinical improvement (median, 15 days vs 16 days) was significant, albeit modest [13]. Besides, more than half of patients, in both study groups, were randomized after 12 days of symptom onset.

In addition to protease inhibitors, such as LVP/RTV, novel nucleotide analogues, such as remdesivir, are also under investigation in clinical trials, as this, has activity against SARS-CoV-2 in vitro and related coronaviruses (including SARS-CoV and MERS-CoV) both in vitro and in animal studies [9, 14, 15]. Also, chloroquine/hydroxychloroquine, have been reported to inhibit SARS-CoV-2 in vitro, although hydroxychloroquine appears to have more potent antiviral activity [7, 9, 15–20]. Use of chloroquine is included in interim guidelines [9–12], and at preliminary data seems to associated with reduced progression of the disease and decreased duration of symptoms [7, 9, 15–20]. Nevertheless, primary experimental data supporting that have not been published [9]. Even, there is a lack of case reports of COVID-19 patients recovering from SARS-CoV-2 infection [21, 22]. We present a confirmed case of COVID-19 from Buga, Valle del Cauca, Colombia, that successful recovered of SARS-CoV-2 infection after receiving chloroquine.

### Case presentation

On March 6, 2020, a 34-year-old Colombian man, from Buga, Valle del Cauca, with class II-obesity was admitted to the emergency department of the Hospital San Jose de Buga, a mid-complexity private institution that historically serves to public health network of Valle del Cauca, for a low-grade fever, chills, fatigue, cough, clear-sputum production, myalgia, arthralgia, rhinitis, adynamia, and weakness. He had become ill on February 29, 2020, a total of 4 days after he had flown to Cali from Madrid, Spain, on March 2, 2020, where he was living since January 7, 2020. He began self-medication with acetaminophen on March 3, 2020. After his arrival at the Cali airport, he travelled by car to Buga, where he stayed until admission to our hospital. He denied contact in Spain with people presenting respiratory symptoms. At the hospital, he was strictly isolated. His close contacts were investigated by the public health authorities and were ruled out.

When the patient arrived, he was alert; heart rate was 82 bpm, blood pressure was 110/60 mm Hg, respiratory rate was 18 breaths/min, the temperature was 36.0 °C. Physical examination revealed no alterations, and the saturation of peripheral oxygen was 97%. His body mass index was 36.42 kg/m^2^. Laboratory findings included mild leukopenia (2.85 × 10^9^ cells/L [reference 3.7–10.1 × 10^9^ cells/L]), absolute lymphopenia (0.762 × 10^9^ cells/L [reference 1.09–2.99 × 10^9^ cells/L]), low monocytes count (0.150 × 10^9^ cells/L [reference 0.3–0.9 × 10^9^ cells/L]), mild erythrocytosis (4.81 × 10^6^ cells/L [reference 4.06–4.69 × 10^9^ cells/μL]), moderate thrombocytopenia (92.7 × 10^9^ cells/L [reference 150.0–450.0 × 10^9^ cells/μL]), and increased C-reactive protein (88.05 mg/L [reference 0–5 mg/L]) (Table 1). At the moment, considering that Valle del Cauca is in an epidemic situation of dengue, this arbovirosis was suspected. Dengue IgM- and IgG-antibodies and non-structural protein 1 (NS1) dengue protein through enzyme-linked immunosorbent assay (ELISA) (93.9% sensitivity, 97.4% specificity) were negative. A peripheral blood smear showed no alterations, except for thrombocytopenia and leukopenia, large platelets and platelets clotting. An initial hemoculture, on the second day of admission, grew only *Staphylococcus auricularis*, with two others taken the same day the grew no organisms. *Staphylococcus auricularis* was considered contamination/colonization.

**Table 1:** Laboratory findings in the patient with COVID-19

Test	Normal values	Date								
		3/6/2020	3/7/2020	3/8/2020	3/9/2020	3/10/2020	3/11/2020	3/12/2020	3/13/2020	3/14/2020
Blood leukocyte count, 10^9^/L	3.7–10.1	*2.850*	*1.930*	*2.930*	*2.870*	*3.390*	*3.690*	*2.350*	*3.360*	3.860
Lymphocyte, %	18.0–48.3	26.70	24.80	30.10	25.40	18.60	21.00	22.40	25.10	22.00
Lymphocyte count, 10^9^/L	1.09–2.99	*0.762*	*0.479*	*0.883*	*0.728*	*0.630*	*0.777*	*0.525*	*0.844*	*0.849*
Neutrophil, %	39.3–73.7	67.50	65.60	63.50	65.60	73.30	69.70	62.90	60.90	66.00
Neutrophil count, 10^9^/L	1.63–6.96	1.920	*1.260*	1.860	1.880	2.490	2.570	*1.480*	2.050	2.550
Neutrophil to lymphocyte ratio (NLR)	0.78–3.53	2.528	2.645	2.110	2.583	*3.941*	3.319	2.808	2.426	3.000
Monocyte, %	0.00–10.0	5.40	8.10	5.95	8.45	7.30	8.76	*13.00*	*11.80*	*10.10*
Monocyte count, 10^9^/L	0.3–0.9	*0.150*	*0.160*	*0.170*	*0.240*	*0.250*	0.320	0.310	0.400	0.390
Eosinophil, %	0.00–7.00	0.00	0.26	0.17	0.22	0.51	0.41	1.23	1.58	1.42
Eosinophil count, 10^9^/L	0–0.5	0.000	0.010	0.010	0.010	0.020	0.020	0.030	0.050	0.060
Basophil, %	0.00–1.00	0.31	1.23	0.26	0.39	0.29	0.10	0.43	0.56	0.49
Basophil count, 10^9^/L	0–0.2	0.010	0.020	0.010	0.010	0.010	0.000	0.010	0.020	0.020
Erythrocyte count, 10^6^/μL	4.06–4.69	*4.810*	4.620	*4.710*	*4.710*	4.650	4.570	4.670	*4.870*	*4.970*
Hemoglobin, g/dL	11.7–18	14.20	13.90	14.10	13.90	13.80	13.60	13.60	14.30	14.50
Hematocrit, %	37.7–53.7	44.50	42.70	43.40	42.90	42.60	41.80	42.60	43.90	45.30
Mean cell volume (MCV), fL	79.0–101.0	92.60	92.50	92.30	91.00	91.60	91.50	91.40	90.10	91.20
Mean cell hemoglobin (MCH), pg	26.0–35.0	29.60	30.20	29.90	29.50	29.80	29.80	29.10	29.40	29.20
Mean corpuscular hemoglobin concentration (MCHC), g/dL	31.0–37.0	31.90	32.60	32.40	32.50	32.50	32.50	31.80	32.70	32.00
Red blood cell distribution width (RDW), μm	11.5–14.5	*10.90*	*10.80*	*10.80*	*10.70*	*10.70*	*10.70*	*10.90*	*10.30*	*10.50*
Platelet count, 10^9^/L	150–450	*92.7*	*98.2*	*107.0*	*122.0*	*147.0*	172.0	196.0	262.0	285.0
Mean platelet volume, Fl	4.5–10	7.710	8.220	8.020	7.310	6.770	7.520	6.950	6.430	6.860
C-reactive protein level, mg/L	0–5	*88.05*	*83.37*	*86.37*	*75.02*	*94.28*	–	*70.44*	*–*	*12.96*
Albumin level, g/L	3.5–5.2	–	–	–	4.0	–	–	–	–	–
Total bilirubin, μmol/L	0.3–1.2	–	–	–	0.52	–	–	–	–	–
Direct bilirubin, μmol/L	0.0–0.5	–	–	–	0.26	–	–	–	–	–
Indirect bilirubin, μmol/L	0.0–0.5	–	–	–	0.26	–	–	–	–	–
Creatinine, mg/dL	0.73–1.18	–	–	0.80	–	–	–	–	–	–
Blood urea nitrogen, mg/dL	8.9–20.6	–	–	*8.14*	–	–	–	–	–	–
Alanine aminotransferase, U/L	0–55	–	–	–	41.4	–	–	–	–	–
Aspartate aminotransferase, U/L	5–34	–	–	–	33.7	–	–	–	–	–
Gamma-glutamyltransferase (GGT), U/L	12–64	–	–	–	55.1	–	–	–	–	–
Alkaline phosphatase, U/L	40–150	–	–	–	34.0	–	–	–	–	–
Lactic acid level, mmol/L	0.5–2.2	–	–	0.95	0.79	–	–	–	–	–
Potassium, mmol/L	3.5–5.1	–	–	–	3.83	–	–	–	–	–
Sodium, mmol/L	136–145	–	–	–	140.0	–	–	–	–	–
Serum chloride, mmol/L	98–107	–	–	–	*109.0*	–	–	–	–	–
Prothrombin time (Seg)	11.7–15.3	–	–	–	12.6	–	–	–	–	–
Partial thromboplastin time (PTT), seg	23.6–34.8	–	–	–	32.8	–	–	–	–	–
INR (International Normalized Ratio)		–	–	–	0.95	–	–	–	–	–
Arterial-blood gases										
pH	7.36–7.44	–	7.422	7.417	*7.449*	–	*7.441*	7.436	–	–
pCO2 (mmHg)	33–40	–	*31.3*	33.4	*30.1*	–	*32.4*	*30.6*	–	–
pO2 (mmHg)	70–80	–	*62.4*	*60.4*	73.9	–	*64.8*	72.6	–	–
HCO3 (mmol/L)	21–27	–	*19.9*	21.0	*20.4*	–	21.6	20.1	–	–
BEb base excess (mmol/L)	− 3 to + 3	–	**− ***3.4*	**− **2.4	**− **2.4	–	**− **1.7	**− **2.9	–	–
SO2%		–	92.0	91.2	94.9	–	92.3	93.6	–	–
Temperature (℃)		–	37.0	37.3	37.0	–	37.0	37.0	–	–
FIO2%		–	–	21.0	21	–	21.0	–	–	–

Italics, altered values

Screenings for common infectious diseases, including those in the FilmArray^®^ Respiratory Panel, multiplex PCR, which was performed on admission day, were all negative (Table 2). Although that, and due to the persistence of fever and respiratory symptoms, community-acquired pneumonia was suspected. Treatment with ampicillin/sulbactam (intravenous 3 g q4h) and clarithromycin (intravenous 500 mg q12h) was initiated.

**Table 2:** Pathogens that were assessed in the patient with COVID-19

Tests	Result
Dengue tests	
NS1 antigen	Negative
IgM	Negative
IgG	Negative
FilmArray respiratory panel, multiplex PCR	
Adenovirus	Negative
Coronavirus 229E	Negative
Coronavirus HKU1	Negative
Coronavirus NL63	Negative
Coronavirus OC43	Negative
Human Metapneumovirus	Negative
Human Rhinovirus/Enterovirus	Negative
Influenza A	Negative
Influenza B	Negative
Parainfluenza 1	Negative
Parainfluenza 2	Negative
Parainfluenza 3	Negative
Parainfluenza 4	Negative
Respiratory syncytial virus	Negative
*Bordetella pertussis*	Negative
*Bordetella parapertussis*	Negative
*Chlamydophila pneumoniae*	Negative
*Mycoplasma pneumoniae*	Negative
HIV-1 and -2 ELISA	Negative
Hemoculture	
*Staphylococcus auricularis*^a^	Positive
Peripheral blood smear	Thrombocytopenia

^a^Two successive hemocultures were negative, suggesting contamination/colonization

**Fig. 1:**
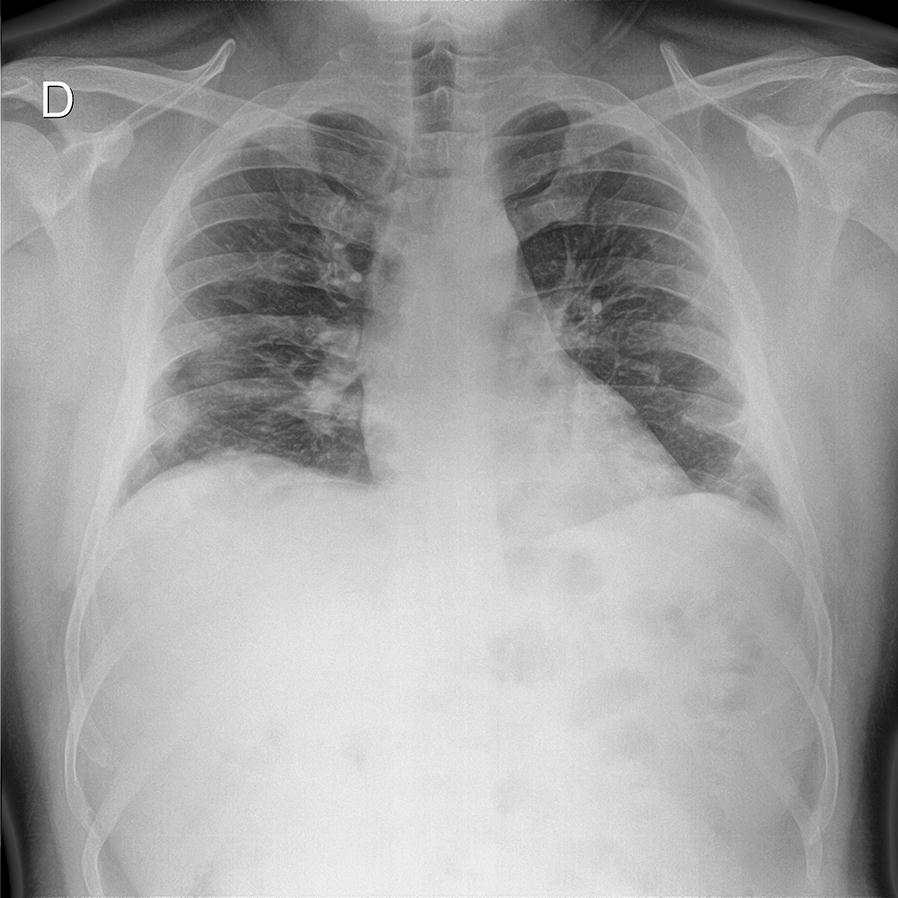
Chest radiograph obtained on admission shows peripheral ground-glass opacities in mid- and lower-third of the thorax

**Fig. 2:**
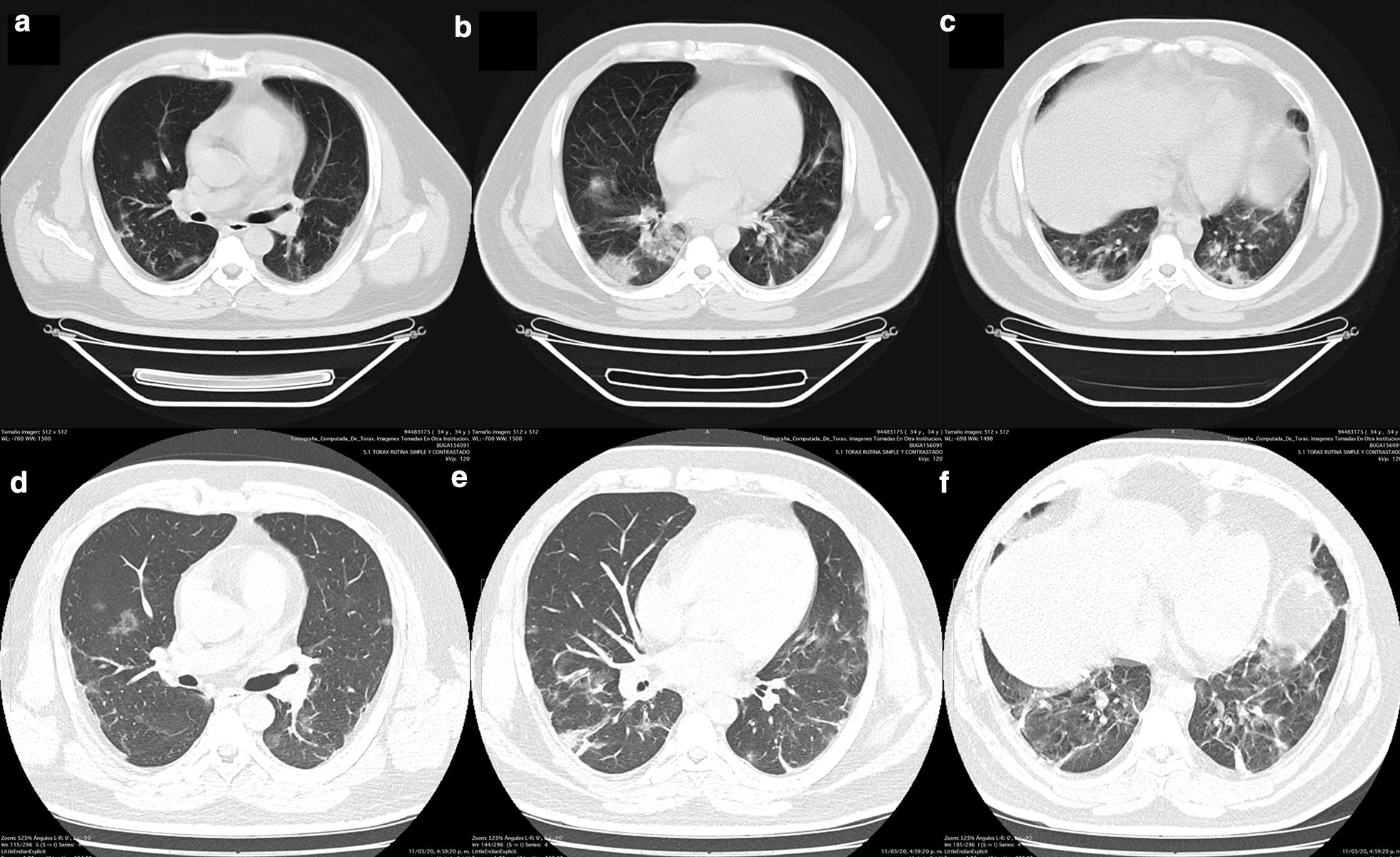
**a**–**c** Chest computed tomography on admission showed peripheral ground-glass opacities with a partial occupation, and also complete consolidation in mid- and lower-third of the thorax. **d**–**f** Chest computed tomography, 4 days later, showed peripheral ground-glass opacities with partial occupation improving, the reversed halo sign is observed, with decreasing in the size of the consolidations

Chest radiographs obtained on admission showed peripheral ground-glass opacities in mid- and lower-third of the thorax (Fig. 1). Chest computed tomography on admission showed peripheral ground-glass opacities with partial occupation and also complete consolidation in mid- and lower-third of the thorax (Fig. 2a–c). We treated the patient with oxygen inhalation, antibiotics, and nutritional support. Blood gas analysis the second day of hospitalization showed that PCO_2_ was 31.3 mm Hg (reference 33–40 mm Hg), PO_2_ was 62.4 mm Hg (reference 70–80 mm Hg), pH was 7.42 (reference 7.36–7.44), HCO_3_ was 19.9 (reference 21–27 mmol/L), base excess − 3.4 (reference − 3 to + 3 mmol/L), and SO2 was 92%, showing a compensated respiratory alkalosis (Table 1). The patient remains stable with no signs of respiratory distress, a saturation of peripheral oxygen was 94%, room air, but with a temperature of 38.4 °C and cephalea, both solved with acetaminophen. At the second day patient also received oseltamivir (orally 75 mg q12h).

At the third day, the lung sounds were decreased at the bases, with a saturation of peripheral oxygen of 95%. His platelet counts improved (107 × 10^9^ cells/μL) (Table 1). His respiratory alkalosis improved partially (Table 1).

Real-time reverse-transcription PCR (rRT-PCR) analysis of the patient’s nasopharyngeal swab specimen indicated SARS-CoV-2 infection on the third day of admission. The test was performed at the Virology Reference Laboratory of the National Institute of Health, Bogotá, Colombia, as recommended by the WHO guidelines, following the protocol Charité, Berlin, Germany [23]. The laboratory confirmed the negative results for Influenza A and B by rRT-PCR. Sequencing of the strain from the patient was not performed.

At the fourth day, it was decided to add chloroquine, phosphate, to the treatment (orally 300 mg, base, q12h) per 10 days plus continuing oseltamivir and antibiotics. At the physical examination he remains stable, with normal respiratory sounds, but continuing with febrile episodes, treated with acetaminophen. His thrombocytopenia continues to improve, and his coagulation times are normal, also his alanine and aspartate aminotransferases, gamma-glutamyltransferase, and albumin (Table 1).

At the fifth day, his platelet counts are almost normal (Table 1), remaining with fever, cough, and leukopenia (including lymphopenia), with no requirements of supplemental oxygen. Lungs physical examination remains normal. The patient presented symptoms of anxiety and trazodone (orally 50 mg) was initiated, improving the next day. His arterial blood gases partially improved (Table 1).

At the sixth day, the platelet counts became normal (Table 1). He presented mild dyspnea during walking to the bathroom. His respiratory conditions, besides that, remains stable. His cough has also improved. A chest computed tomography on this day showed peripheral ground-glass opacities with partial occupation improving, the reversed halo sign is observed, with decreasing in the size of the consolidations (Fig. 2d–f). A psychologist assessed him, recommending preventing education for his return to home with family.

At the seventh day his clinical condition had improved, with no fever, no dyspnea, no thrombocytopenia, with normal arterial-blood gases and improvement of the C-reactive protein levels, but remaining with leukopenia and lymphopenia (Table 1).

At the eighth day the leukopenia improved (Table 1), remaining with fever (completing 72 h afebrile), with a heart rate of 90 bpm, blood pressure was 120/80 mm Hg, respiratory rate was 20 breaths/min, the temperature was 36.2 ℃. Physical examination revealed no alterations, and the saturation of peripheral oxygen was 94%. At this day, chloroquine and oseltamivir were discontinued after completed 5 days of treatment.

At ninth day, the leukocyte counts became normal, with no other significant clinical findings, except for a mild elevation of the C-reactive protein (Table 1). His clinical condition significantly improved. There is no fever nor cough. A control sample for rRT-PCR for SARS-CoV-2 took this day was negative. The patient is discharged this day. He will remain isolated at home for 14 days at a separated room, with no contact with relatives and proper room ventilation, use of face mask, and instructions for regular hand washing, among other measures, and under close medical follow up. Nine days after he was discharged, he remains in good condition, no additional symptoms, and considered successfully recovered.

## Discussion

European countries are currently experiencing the largest outbreak of COVID-19 in the World. Italy reported a cumulated of 63,927 cases up to March 23, 2020. Just 21.6% fewer cases than China (81,496) at that moment. Italy had the highest number of COVID-19 cases reported for Europe on March 28, 2020 [24–27]. But for April 23, 2020, Spain, with 213,024 cases had the highest number in the continent, and second in the world after United States (866,646).

For the moment our patient arrived in Colombia, Spain was the second most affected European nation, after Italy. Both countries spreading cases all over the world, including Latin America. Our case, came from Madrid, Spain, in a critical moment of the outbreak, where that capital city, presented the highest number of cases [28, 29]. This was the second diagnosed case in Colombia, with the fortune to be located in a small municipality of Valle del Cauca department, Buga, with only 114,041 inhabitants.

As expected, this patient presented fever, cough, dyspnea, myalgia, fatigue, headache, among other reported clinical manifestations [30–36]. He only presented as a risk factor, class II-obesity, and was managed isolated, without requiring intensive care unit (ICU), nor mechanical ventilation. C-reactive protein, lymphopenia, and leukopenia were also presented, but no hypoalbuminemia, LDH, hepatic enzymes, bilirubin, nor creatinine were altered [36]. Also, his imaging findings were consistent to those reported in the literature, bilateral multilobar ground-glass opacification with a peripheral distribution, as well as, the presentation of consolidative opacities [37]. This patient did not present complications and evolved clinically well.

Up to date, there is a lack of recommendations for the use of any antiviral drug in the treatment of COVID-19 [6–9]. Then, expert recommendations are rapidly assessing potential therapeutical drugs that may help in the management of patients with SARS-CoV-2 infection, including chloroquine, as currently reported in many emerging interim guidelines [9–12]. In Colombia, the Ministry of Health and the Colombian Association of Infectious Diseases [38], have considered the possible use of chloroquine for COVID-19 patients, in those hospitalized under close medical observation, as was our case.

Both chloroquine and hydroxychloroquine, have an excellent safety record and are well distributed throughout the whole body after oral administration, especially in acidic compartments such as lysosomes and inflamed tissues [39]. Gastrointestinal responses, such as vomiting and diarrhoea, are the most common adverse effects of these two drugs, but in our case, he did not present them [39, 40]. The current evidence suggesting the use of chloroquine, as we did, is only based on in vitro studies. At the moment, multiple randomized controlled trials are being conducted to test the effect of chloroquine in treating COVID-19 [41]. Nowadays, The effective concentrations (EC_90_) of chloroquine for SARS-CoV-2 in Vero E6 cells is 6.90 μM [15], which is clinically achievable, well-tolerated in patients with rheumatoid arthritis and potentially applicable to COVID-19 patients, as we did. Our patient received 5 days of chloroquine per 5 days at 600 mg per day (divided into two doses, 300 mg/day base). That is the dose recommended by different interim guidelines [42]. According to a consensus statement from a multicenter collaboration group in China, chloroquine phosphate 500-mg twice daily in tablet form for 10 days may be considered in patients with COVID-19 pneumonia [43, 44]. Although optimal dosing and duration of these drugs for the treatment of COVID-19 are unknown, its use is being reported in different COVID-19 cases, as monotherapy or combined [7, 8, 15–20, 39–41, 43]. A recent structural and molecular modeling study showed that chloroquine binds sialic acids and gangliosides with high affinity, and the S protein of SARS-CoV-2 uses the ACE-2 receptor for entry, but also sialic acids linked to host cell surface gangliosides. Then, the identification of this new mechanism of action of chloroquine supports its potential use the SARS-CoV-2 infection [45].

The first intended use of chloroquine in our patient was as monotherapy, but unintentionally for SARS-CoV-2, he also received 5 days of an erythromycin analogue, clarithromycin [46]. The erythromycin analogue, azithromycin, has been reported in one small study in combination with reported that hydroxychloroquine reducing the detection of viral RNA in upper respiratory tract specimens compared with a non-randomized control group, but did not assess clinical benefit [47, 48].

Hydroxychloroquine and azithromycin are associated with QT prolongation and caution are advised when considering these drugs in patients with chronic medical conditions (e.g. renal failure, hepatic disease) or who are receiving medications that might interact to cause arrhythmias [47, 48]. In the case of chloroquine, it appears that fusion and un-coating blockade, by lysosomal alkalization [49, 50]; interaction with the ACE2 receptor [49, 50]; and immuno-modulation act as a mechanism to control SARS-CoV-2 infection [42].

Our patient, instead of azithromycin, received clarithromycin. Both inhibit protein synthesis in susceptible organisms (e.g. bacteria) by binding to the 50S ribosomal subunit. Clarithromycin is several-fold more active in vitro than erythromycin against gram-positive organisms, while azithromycin is 2- to 4-fold less potent [46]. Clarithromycin has a longer serum half-life and better tissue penetration than erythromycin, allowing twice-a-day dosing for most common infections [46]. In addition to common bacteria, azithromycin and clarithromycin have demonstrated to be also active against some unexpected pathogens (e.g., *Borrelia burgdorferi*, *Toxoplasma gondii*, *Mycobacterium avium* complex, and *M. leprae*), and maybe also for SARS-CoV-2 [46]. The efficacy of clarithromycin has been examined against H5N1 highly pathogenic and H7N9 low pathogenic avian influenza virus infections in cynomolgus monkeys, showing viral suppression and clinical improvement [51]. A study assessed the efficacy and safety of a clarithromycin-naproxen-oseltamivir combination for the treatment of serious influenza, also showing good results, reducing both 30- and 90-day mortality and length of hospital stay [52]. Then, the antiviral activity and clinical studies with chloroquine or hydroxychloroquine, azithromycin or clarithromycin, as monotherapy or especially in combination, should be specially assessed in the immediate future.

Although that just based in one case, we cannot recommend the use of these drugs, our patient improved significantly, and his clinical manifestations ceased, including becoming negative for the SARS-CoV-2 infection, as observed in the rRT-PCR test. Also, we cannot be sure of the antiviral effect of chloroquine and clarithromycin, but both drugs were well tolerated, easy to administrate, and specifically, in our case, they were not associated with adverse effects.

Finally, this and other COVID-19 cases, are urgently demanding results from clinical trials that support evidence-based therapeutical approaches to this pandemic.

## Limitations

Our case has different limitations. Colombia will need to have sequencing and phylogenetic studies that would be useful as its isolates may diverge from other SARS-CoV-2 isolates or strain, that even, would be related to clinical evolution and outcomes, as this case. Even more, we are not performing yet quantitative RT-PCR and measurements of the viral load, that would also be correlated with clinical evolution, and maybe immune and therapeutic responses. Finally, no results from good trials are available that support yet the use of chloroquine and azithromycin, nevertheless, in this case, as probably in others, the clinical evolution was satisfactory.

## Data Availability

Copy of the clinical data of the patient is available.
